# Conservation of human NMDA receptor subunits and disease variants in zebrafish

**DOI:** 10.21203/rs.3.rs-7151578/v1

**Published:** 2025-07-30

**Authors:** Erica R. Nebet, Christieann Aprea, Josiah D. Zoodsma, William Raab, Howard I. Sirotkin, Lonnie P. Wollmuth

**Affiliations:** Stony Brook University; Stony Brook University; Stony Brook University; Stony Brook University; Stony Brook University; Stony Brook University

**Keywords:** GRIN genes, ClinVar, gnomAD, sequence alignments, disease-associated variants, N-terminal domain, ligand-binding domain, transmembrane domain, C-terminal domain, ion channel

## Abstract

**Background:**

NMDA receptors (NMDARs) are widely expressed, glutamate-gated ion channels that play key roles in brain development and function. Variants have been identified in the *GRIN* genes encoding NMDAR subunits that are linked to neurodevelopmental disorders, among other manifestations. Zebrafish are a powerful model to study brain development and function given their rapid development and ease of genetic manipulation. As a result of an ancient genome duplication, zebrafish possess two paralogues for most human NMDAR subunits. To evaluate the degree of conservation between human NMDAR subunits and their respective zebrafish paralogues, we carried out detailed *in silico* analyses, with an emphasis on key functional elements. To further assess the suitability of zebrafish for modeling NMDAR-associated neurodevelopmental disorders, we analyzed the conservation of positions with identified missense variants.

**Results:**

We find that the human NMDAR subunits are generally well conserved across zebrafish paralogs. Moreover, variants classified as pathogenic and putatively pathogenic are highly conserved, reflecting the importance of key protein regions to neurotypical receptor function. Positions with putatively benign and benign variants are less conserved. Across NMDAR domains, the transmembrane domain is most highly conserved, followed by the ligand-binding domain, which maintains conservation of amino acids that participate in the binding of ligands. The N-terminal domain is less well conserved but aligned homology models show high degrees of structural similarity. The C-terminal domain is the most poorly conserved region across zebrafish paralogs, but certain key regions that undergo phosphorylation, palmitoylation, and ubiquitylation as well as protein-binding motifs are better conserved.

**Conclusions:**

Our findings highlight a strong conservation of human NMDAR subunits in zebrafish, with some exceptions. The ligand-binding domain, the transmembrane domain forming the ion channel and the short polypeptide linkers that connect them are highly conserved. The N- and C-terminal domains are less conserved but functional motifs in general, except for the Zn^2+^ binding site in GluN2A paralogues, are more highly conserved relative to the entire domain. Overall, our findings support the utility of zebrafish as a model for studying neurodevelopment and disease mechanisms and provide a template for rigorously considering the relationship between human and zebrafish paralogues.

## BACKGROUND

Rapid cell-to-cell signaling in the nervous system occurs at specialized structures called synapses. At these sites, the electrical activity of the upstream or presynaptic neuron is converted into the release of a chemical neurotransmitter. This is then converted back into an electrical signal in the postsynaptic neuron by specialized ion channels that are gated by a neurotransmitter, initiating downstream biological signaling cascades. Glutamate, the primary excitatory neurotransmitter in the brain, activates a variety of receptors that are essential for synaptic transmission and plasticity. Glutamate-gated ion channels play fundamental roles in signaling in both the developing and mature nervous systems (1–9).

The NMDA receptor (NMDAR) is a prominent glutamate-gated ion channel subtype (1, 2). Due to its distinct biophysical properties and unique ionotropic and non-ionotropic signaling mechanisms, it contributes to a wide array of nervous and non-nervous system functions (2, 10–14). Numerous missense, frameshift, and nonsense mutations (or variants) have been identified in the genes that encode NMDAR subunits and are associated with neurodevelopmental disorders, including autism spectrum disorders (ASD), intellectual disability, developmental delay, seizures, and schizophrenia (15–23). However, how these variants lead to clinical phenotypes is poorly understood.

Zebrafish are a powerful model organism to study neurodevelopmental phenotypes and disorders, including those linked to NMDARs (19, 24–32). Zebrafish develop rapidly and share fundamental developmental and signaling pathways with humans and other vertebrates (33); they are transparent, allowing for dynamic and non-invasive observations of developmental and cellular processes; and they display robust behaviors that are easily assayed at early larval stages. Moreover, larval zebrafish readily take up small molecules from their environment, enabling non-invasive and high-throughput readout of nervous system function in the context of behavioral drug screens (34–36).

**Figure 1:**
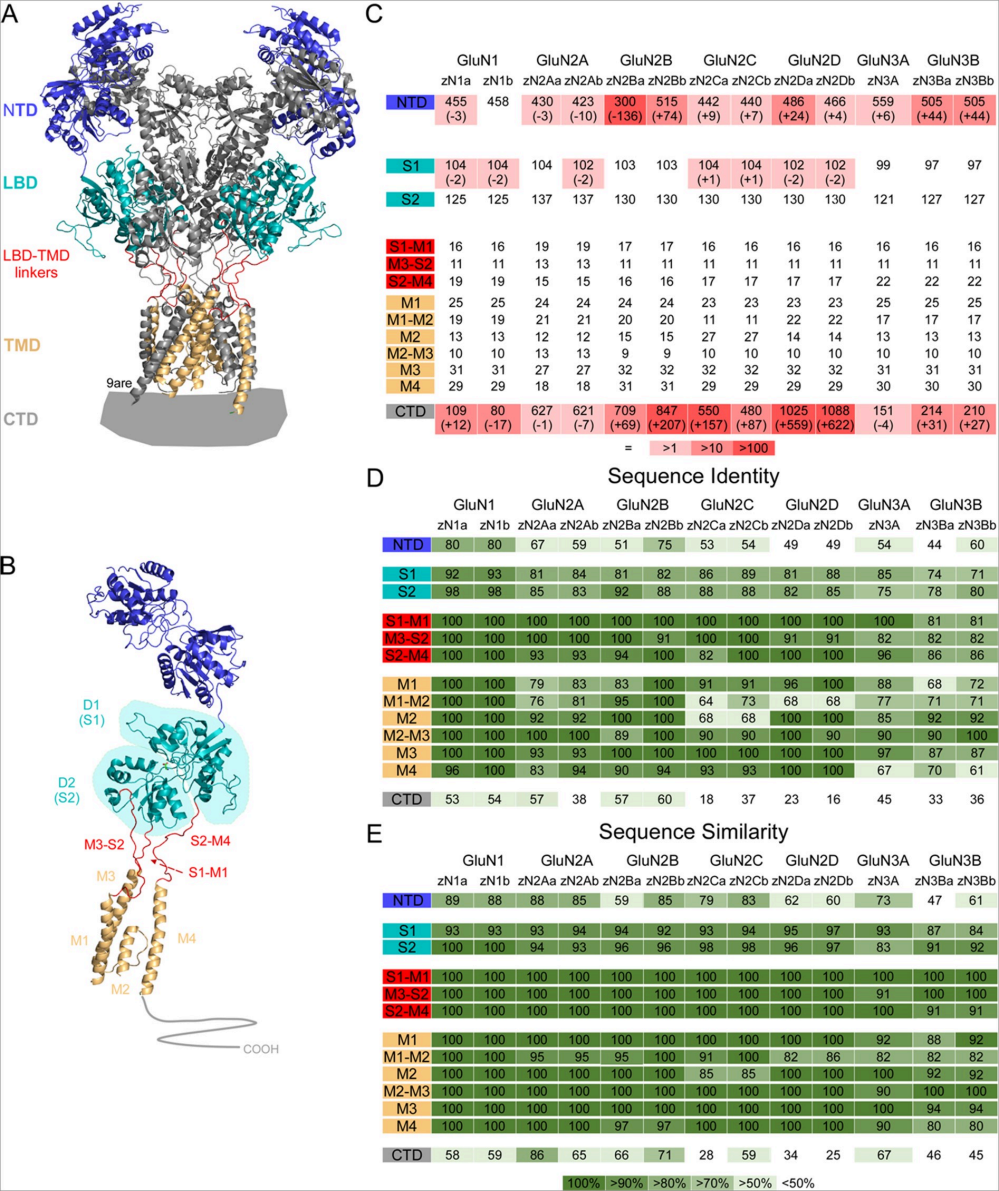
Conservation of human NMDAR subunits in zebrafish **(A)** NMDARs are composed of two obligatory GluN1 and typically two GluN2A-D subunits. The tetrameric complex consists of four domains: extracellular N-terminal (NTD) and ligand-binding (LBD) domains; a transmembrane domain (TMD); and an intracellular C-terminal domain (CTD). GluN1 subunits are colored dark blue (NTD), teal (LBD), red (LBD-TMD linkers), light orange (TMD), and gray (CTD); and GluN2B subunits gray. 9are (82) **(B)** Topology of an individual GluN1 subunit. LBD lobes (D1 and D2) are shown; D1 is mainly comprisedof S1 and D2 of S2. The ion channel is formed by 3 transmembrane segments, M1, M3, and M4, and an intracellular M2 pore loop. LBD-TMD linkers are S1-M1 (or D2-M1), M3-S2 (or D2-M3), and S2-M4 (or D1-M4). **(C)** NMDAR subunits are separated into 13 structural subdivisions: NTD, LBD (S1 & S2), LBD-TMD linkers (S1-M1, M3-S2, & S2-M4), TMD (M1, intracellular M1-M2 linker, M2 loop, M2-M3 linker, M3, & M4), and CTD. Amino acid length of each zebrafish subdivision is indicated. Those sharing amino acid length with their human orthologues are indicated in white, while increasingly darker pink indicates those with increasingly greater amino acid differences (greater (+) or fewer (−)). **(D-E)** NMDAR subunits are grouped as in **(C)**. Homology between human and zebrafish NMDAR subunit paralogues is reported in amino acid identity **(D)** or similarity **(E)**. Heat map with darker green indicating greater percent of homology.

NMDARs are heterotetramers composed of two obligatory GluN1 (encoded by *GRIN1*) subunits. They are expressed as either diheterotetramers with two GluN1 and two GluN2 (*GRIN2A-D*) subunits or triheterotetramers of some combination of GluN2 and/or GluN3 (*GRIN3A-B*) subunits (2) (Fig. 1A). These NMDAR subunits are differentially expressed over the course of neurodevelopment, with GluN2A, GluN2B, and GluN2D being highly expressed during early development, while GluN2C, GluN3A, and GluN3B expression increases into adulthood (1). Moreover, NMDARs display distinct functional and pharmacological properties based on their GluN2 or GluN3 composition (2). Each subunit consists of four principal domains: an N-terminal domain (NTD), a ligand-binding domain (LBD), a transmembrane domain (TMD) that forms the ion channel, and a C-terminal domain (CTD) (Fig. 1B).

Disease-associated variants (DAVs) in *GRIN1, GRIN2A, GRIN2B*, and *GRIN2D* have been identified in pediatric patients with neurodevelopmental disorders (15–18, 21). These disorders often present with multiple comorbidities, reflecting overlapping neurodevelopmental phenotypes (37). Generally, patients with DAVs in *GRIN1* present with developmental delay and intellectual disability, though a large proportion of patients also experience seizures (15). In contrast, *GRIN2A* and *GRIN2D* are predominantly associated with seizure disorders (16, 18). *GRIN2B* is a high-risk gene for autism spectrum disorder (ASD) (17, 38, 39), as are *GRIN1* and *GRIN2A* (40–42). Still, patients with such neurodevelopmental disorders generally experience a variety of both central and peripheral nervous system symptoms (43–46).

NMDA receptors are highly conserved across species (47). Due to an ancient genome duplication event, zebrafish retain two paralogues for most NMDAR genes (e.g., *grin1a & grin1b*) (24, 48). Patch clamp electrophysiology experiments show that zebrafish paralogues display similar functional properties to each other and to their mammalian orthologues (27, 28). Nevertheless, it remains unclear how well the human NMDAR genes, including positions with identified missense variants, are conserved in zebrafish. To address this, we investigated NMDAR conservation in zebrafish by taking advantage of amino acid sequence alignments (28) and assessed the conservation of all human NMDAR subunits and zebrafish paralogues. We also compared the conservation of positions with missense variants identified in human patients for NMDAR subunits expressed during early neurodevelopment. Finally, we investigated the conservation of critical functional regions within each structural domain. Through this analysis, we define the extent to which critical functional components and regions, with relevance to disease, are conserved across zebrafish NMDARs, providing a template to use zebrafish as a model to study NMDARs in neurodevelopment and disease pathology.

## METHODS

### NMDAR sequences and sequence alignments

Amino acid sequences for human genes are (UniProt with protein IDs): GluN1 (Q05586–5; NTD splice isoform, NR1–3), GluN2A (Q12879–1), GluN2B (Q13224), GluN2C (Q14957), GluN2D (O15399), GluN3A (Q8TCU5), and GluN3B (O60391).

For mouse subunits: mGluN1 (P35438), mGluN2A (P35436), mGluN2B (Q01097), mGluN2C (Q01098), mGluN2D (Q03391), mGluN2A (A2AIR5), and mGluN3B (Q91ZU9).

For zebrafish paralogs: zGluN1a (F1R366), zGluN1b (Q6ZM67), zGluN2Aa (A0A8M9PXD6), zGluN2Ab (F1QDE5), zGluN2Ba (A0A8M9Q0I1), zGluN2Bb (A0A8M1NH89), zGluN2Ca (E7FH62), zGluN2Cb (A0A8M3B7K5), zGluN2Da (I3NI77), zGluN2Db (E7F3Z4), zGluN3A (A0A8M3AWH5), zGluN3Ba (A0A8M9P9F6), and zGluN3Bb (A0A8M6Z151).

Breakpoints for NMDAR subunit domains and subdomains are based on structures 6WHR (rat GluN1-GluN2B) and 7EU7 (human GluN1-GluN2A) (49, 50).

Human and zebrafish protein sequences were aligned and analyzed for amino acid identity and similarity using Align Sequences Protein BLAST (blastp), which is publicly available through the National Center for Biotechnology Information (NCBI) or EMBOSS Needle Pairwise Sequence Alignment (PSA). For each NMDAR subunit, the zebrafish paralogs were individually aligned to the human protein. Amino acid similarity is defined based on the BLOSUM 62 substitution matrix. For the NTD, LBD, and TMD, these alignments were generally straightforward as the sequences do not diverge greatly in length. In contrast, the CTD is quite large, with the zebrafish sequences generally larger than human. For these alignments, we entered the entire sequence into the alignment tool and used the given result, though this led to a fragmentation of the CTD such that certain portions of the sequence were aligned while others were not. We used the resulting domain-specific conservation values to calculate the conservation of the total protein.

### Variants analysis

Human NMDAR variants were identified using ClinVar and gnomAD (Table 2).

#### ClinVar.

Results for each gene were downloaded on September 28th, 2023, using ClinVar’s bulk download option (https://www.ncbi.nlm.nih.gov/clinvar/). Genomic variants that did not result in protein coding changes were removed from the analysis. In the case of *GRIN3A*, protein coding changes in the PPP3R2 gene, a single exon gene whose coding sequence resides within the genomic region of *GRIN3A* and is downloaded alongside *GRIN3A* due to this, was also removed. In cases where multiple protein coding annotations exist for the same variant, only the annotation fitting the canonical transcript was retained.

#### GnomAD:

Genes were searched for by name on gnomAD v4.0.0 (https://gnomad.broadinstitute.org/) and missense, insertion, deletion, and loss-of-function variant information was exported on September 28th, 2023 as comma separated value (CSV) files for each gene. HGVS annotation (protein change) was converted to letter-number-letter annotation.

Gene variants from ClinVar and gnomAD were merged using R studio 2023.06.1 Build 524 based on the letter-number-letter annotation. The resulting merged file was checked for redundant information or variant duplicates, which were removed.

Missense variants were extracted and grouped by pathogenicity as pathogenic, putatively pathogenic, complex, putatively benign, and benign variants based on the criteria in Table 2. Assessment of conservation of positions with identified human variants was performed using the generated sequence alignments.

### NMDAR domain-specific analysis

To further refine the relationship between human and zebrafish NMDAR proteins, we analyzed the TMD, LBD-TMD linkers, LBD, NTD, and CTD of GluN1, GluN2A, GluN2B, and GluN2D for conservation of key structural and functional features.

#### Transmembrane domain (TMD) and ligand-binding domain-transmembrane domain (LBD-TMD) linkers.

Key regions involved in channel gating, Mg^2+^ block, and Ca^2+^ flux were identified as described in the literature and analyzed for conservation (51–57).

#### Ligand-binding domain (LBD).

Key regions and specific amino acids involved in Van-der-Waals interactions with the α-carbon of the ligand, interactions with the α-amino group, electrostatic interactions with the α-carboxyl group, and interactions with the amino acid side chain were identified as described in Ramos-Vicente et. al. 2021 and analyzed for conservation (47).

#### N-terminal domain (NTD).

Key amino acid positions required for the binding of zinc and polyamines were identified via a literature search and their conservation assessed (2, 58, 59). We also examined the conservation of putative N-linked glycosylation sites (60). To generate homology models of the zebrafish NTDs, we used the Template function in SWISS-MODEL (61, 62). As reference structures, we used rodent NTD structures 5TQ0 (GluN1-GluN2A in the presence of EDTA, no bound Zn^2+^ for GluN1 and GluN2A), 5TPZ (GluN1-GluN2B apo state for GluN1 and GluN2B), and 5TPW (GluN1-GluN2A with Zn^2+^ in complex with GluN2A) (58). Sequences for human (hGluN1, hGluN2A, and hGluN2B) or zebrafish (zGluN1a, zGluN1b, GluN2Aa, GluN2Ab, GluN2Ba, GluN2Bb) NTDs were entered individually into SWISS-MODEL to generate homology models. The subsequent models were then loaded into PyMole (human and zebrafish paralog) and aligned. The resultant root mean square deviation (RMSD) is reported.

#### Carboxy-terminal domain (CTD).

CTD-specific conservation in zebrafish was assessed for the GluN1, GluN2A, and GluN2B subunits. GluN2D was excluded from this analysis due to the poor conservation of the CTD in these paralogs relative to human. To assess functional relevance, we focused on post-translational modifications (PTMs), specifically phosphorylation sites (serine, threonine, tyrosine), ubiquitylation sites (lysine), and palmitoylation sites (cysteine), using a combination of experimentally validated data from PhosphoSitePlus (PSP) and published literature (2, 11). Phosphorylation sites with identified kinases or well-characterized functional roles were classified as ‘kinase-regulated phosphorylation sites.’ The remaining phosphorylation sites identified in PSP were defined as ‘phosphoproteomic-identified sites.’ Note, sites in PSP (based on rodents) that did not have a phosphorylation site in humans were excluded from our analysis. In addition, known linear protein-binding motifs were annotated and analyzed for conservation (2, 11). To assess conservation of protein-binding motifs in the CTDs, we categorized sites as either short linear motifs (SLiMs) or broader interaction ‘regions’. SLiMs were defined as experimentally validated, compact sequence elements (typically < 15 residues) that mediate specific and direct interactions with known protein partners. Broader regions were defined as extended sequences (> 15 residues) shown to directly bind proteins in biochemical or structural studies but lacking a discrete consensus sequence. All motifs were curated from published literature where discrete sequences were experimentally validated per motif (11, 63–80), and sequence conservation was examined.

## RESULTS

Our goal is to compare the structural similarity of zebrafish NMDAR (zGluN) paralogues to human NMDARs (GluN), especially regarding human variants. Since mice are a common animal model (81), as a reference-point for NMDAR gene conservation, we initially made global comparisons of both mouse and zebrafish NMDAR subunits to human.

### Global comparison of mouse and zebrafish orthologues to human NMDARs

NMDARs are heterotetramers composed of two obligate glycine-binding GluN1 subunits and two glutamate-binding GluN2 subunits or a combination of GluN2 and glycine-binding GluN3 subunits (1, 2) (Fig. 1A,B). Glycine and glutamate act as co-agonists for GluN2-containing receptors, with binding of both required for receptor activation, while GluN3-containing receptors are activated by glycine binding. As an initial assessment of the structural similarity between NMDAR orthologues, we made global sequence alignments using the NCBI blastp or EMBOSS PSA tools. Not surprisingly, given evolutionary proximity, the mouse subunits are highly conserved relative to human: GluN1 (97% identity & similarity), GluN2A (95% identity; 98% similarity), GluN2B (98% identity; 99% similarity), GluN2C (88% identity; 90% similarity), GluN2D (97% identity & similarity), GluN3A (92% identity; 95% similarity), and GluN3B (77% identity; 83% similarity) (Table 1).

On the other hand, the zebrafish paralogues show reduced conservation. The obligatory GluN1 subunits are the most highly conserved, both in terms of identity (≥ 84%) and similarity (≥ 89%) (Table 1). The other subunits show various degrees of identity and similarity: GluN2A (≥ 58% identity; ≥79% similarity), GluN2B (≥ 65% identity; ≥73% similarity), GluN2C (≥ 50% identity; ≥64% similarity), GluN2D (≥ 43% identity; ≥54% similarity), GluN3A (63% identity; 78% similarity), and GluN3B (≥ 54% identity, ≥ 62% similarity) (Table 1). These subunits, thus, retain ≥ 50% identity with respect to the human protein, except for zGluN2Da and zGluN2Db, which only share 43% and 46% sequence identity, respectively (Table 1). With respect to the zebrafish paralog pairs, there are instances in which one paralog is better conserved than the other. The GluN1 paralogs are comparably conserved (Table 1). However, the zGluN2Aa paralog is more highly conserved than zGluN2Ab while the zGluN2Bb shows increased conservation relative to zGluN2Ba (Table 1). The GluN2C, GluN2D, and GluN3B paralogs are conserved to similar extents (Table 1).

Nevertheless, despite lower conservation relative to mouse, zebrafish offer many technical advantages (see Background). In addition, what is critical is the conservation of specific structural and functional domains and subdomains.

Numbers indicate the percent of mouse or zebrafish paralog conservation with respect to the human protein – ‘Id.’ is identity and ‘Sim.’ is similarity. The zebrafish a and b paralogs are indicated for each protein. GluN3A has only a single paralog in zebrafish. For the mouse and zebrafish protein length, numbers in parentheses indicate the difference in amino acid number relative to human.

### Conservation of specific structural elements in zebrafish paralogues

For more rigorous comparisons, we examined the conservation within individual subunit domains and subdomains (Fig. 1). The structure of NMDARs, like all iGluRs, consists of 4 domains that are intrinsic to each individual subunit (Fig. 1A,B): the extracellular NTD and LBD; the TMD forming the ion channel as well as the linkers that connect the LBD to the TMD, the LBD-TMD linkers; and the intracellular CTD (2, 53). Within individual subunits, the LBD is formed by two disparate sequences, S1 and S2, that fold up into the D1 and D2 lobes in the 3-dimensional structure (83, 84). In general, D1 is made up primarily of S1 and D2 of S2, but there is considerable swapping between lobes. The TMD is formed by three transmembrane segments, M1, M3, and M4, and an M2-pore loop. The LBD-TMD linkers are referred to as either S1-M1, M3-S2, and S2-M4 (linear sequence) or D2-M1, M3-D2, and D1-M4 (3-dimensional structure). This distinction is important functionally since D1 and D2 undergo different movements that are translated differently to the TMD (85, 86). Nevertheless, we will use the S1-M1, M3-S2, and S2-M4 designation for ease of comparison to structures.

We began by dividing the zebrafish paralogues into their respective domains and subdomains based on our breakpoints (see Methods). Interestingly, the amino acid sequences surrounding these breakpoints were well conserved in zebrafish – the majority were identical (not shown). This allowed for ease of division of the zebrafish proteins into their respective domains and subdomains.

As an initial assessment of the structural similarity between human and zebrafish NMDARs, we made detailed sequence alignments using the NCBI blastp or EMBOSS PSA tools (Table 1, Fig. 1). In terms of length of domains, the zebrafish protein sequence for GluN1, GluN2, and GluN3 subunits was identical to the human sequence for the TMD (M1, M1-M2, M2, M2-M3, M3, & M4) and LBD-TMD linkers (S1-M1, M3-S2, & S2-M4) (Fig. 1C). The LBD (S1 & S2) was also quite similar in length, with no changes in the length of S2 across the paralogs and only slight increases or decreases in amino acid number in the S1 of zGluN1a, zGluN1b, zGlun2Ab, zGluN2Ca, zGluN2Ca, zGluN2Da, and zGluN2Db (Fig. 1C). The NTD and CTD were less conserved in terms of size, wherein almost every zebrafish paralog has a different number of amino acids compared to the human. This is especially notable for the GluN2B NTD which either lacks 130 amino acids (zGluN2Ba) or has an additional 74 amino acids (zGluN2Bb) as well as for the CTD where, for example, zGluN2Da and zGluN2Db have a CTD that is more than twice as long as that in the human protein (Fig. 1C).

Number of positions with missense variants that have been identified in each subunit per designation. Percentages report the proportion of the total protein amino acids at which such variants have been identified.

We used these sequence alignments to assess the degree of amino acid identity and similarity between domains and subdomains (Fig. 1D,E). The obligatory GluN1 subunit showed the highest degree of conservation. The TMD and LBD-TMD linkers are 100% conserved. The LBD is also well conserved with S1 and S2 showing ≥ 92% identity and ≥ 93% similarity. The NTD is somewhat less conserved (≥ 80% identity, ≥ 88% similarity), as is the CTD (≥ 53% identity; ≥58% similarity).

This trend of conservation across NMDAR subunits – the LBD and TMD are most highly conserved and the NTD and CTD less so – is maintained in the GluN2 and GluN3 subunits (Fig. 1D,E). Generally, the GluN2 subunits maintain a higher sequence identity than the GluN3 subunits. Overall, this sequence analysis suggests that the LBD and TMD are highly conserved whereas the NTD and CTD show less conservation.

### Categorizing the pathogenicity of missense variants

NMDARs with missense, frameshift, and nonsense variants have been identified in pediatric patients with neurodevelopmental disorders (21, 22, 87). We therefore wanted to assess the extent to which variants are conserved in zebrafish to aid in guiding future studies. For this analysis, we focused solely on missense variants. While nonsense and frameshift mutation can be modelled in various ways, such as by mutating a portion of the protein to induce a loss-of-function, the conservation of the specific protein sequence is somewhat less relevant. In contrast, the single amino acid change associated with missense variants is critical to dissecting how such variants alter receptor function and lead to disease phenotypes.

Using publicly available datasets of NMDAR variants from ClinVar and gnomAD, we identified missense variants in all NMDAR subunits (see Methods). We first categorized these variants with regards to their pathogenicity (Table 2). ClinVar reports variants that have been identified in patients with symptomatic disease (88–90), while gnomAD reports variants identified in the healthy population (91). Thus, in general, we define variants present in ClinVar but absent in gnomAD as pathogenic while those with the opposite reporting as benign. With this approach, pathogenic and putatively pathogenic variants were identified in GluN1, GluN2A, GluN2B, and GluN2D while minimal to no such variants were identified in GluN2C, GluN3A, and GluN3B (Table 2). Notably, these genes with known pathogenic variants are expressed early in development, contributing to developmental processes while their dysfunction often leads to disease phenotypes (21, 87). On the other hand, GluN2C, GluN3A, and GluN3B have no pathogenic and a small number of putatively pathogenic variants (Table 2). We therefore focus solely on GluN1, GluN2A, GluN2B, and GluN2D for further analysis.

### Pathogenic and putatively pathogenic variant positions are well conserved in zebrafish

**Figure 2:**
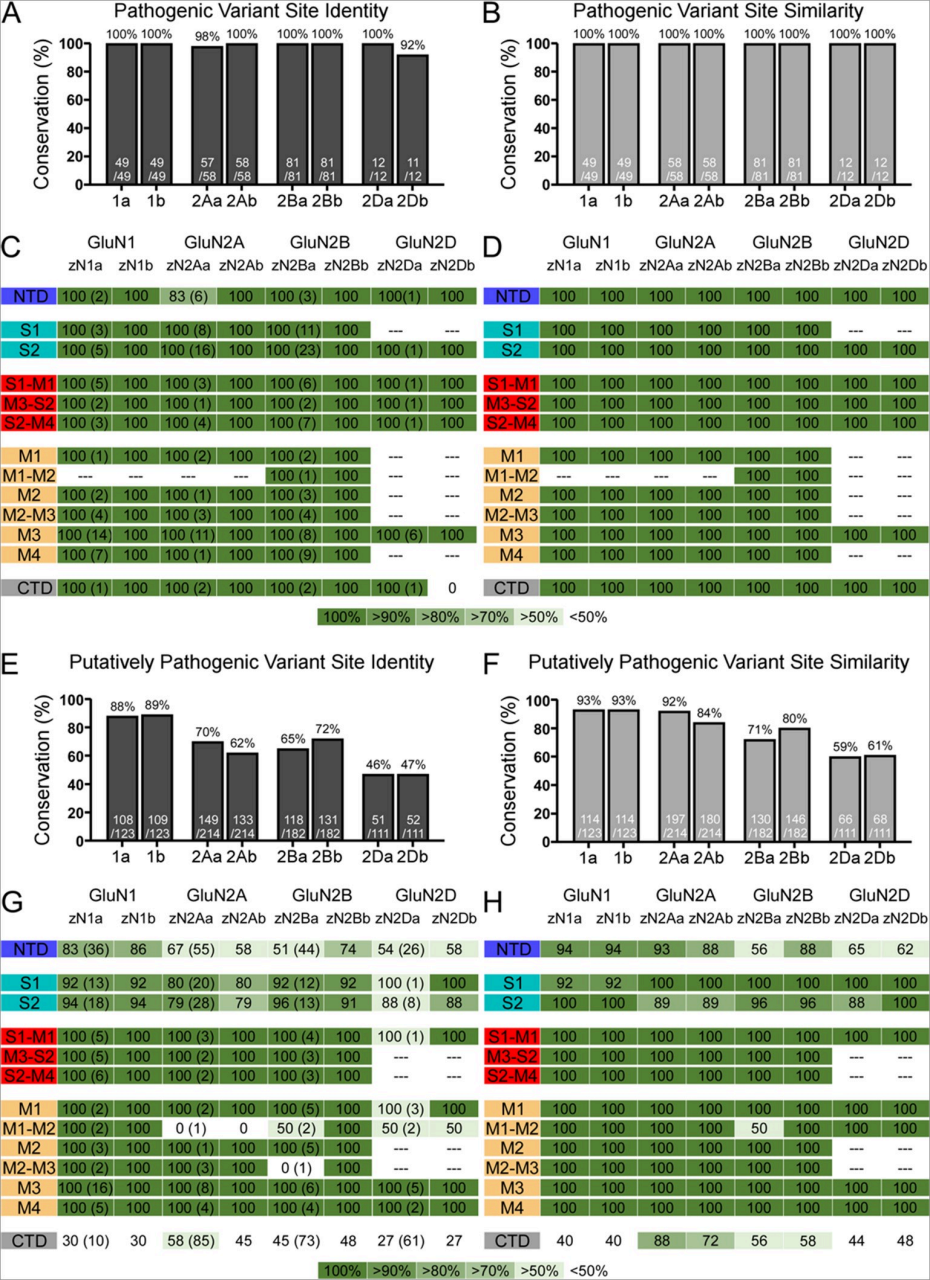
Conservation of positions with pathogenic and putatively pathogenic missense variants **(A-B)** Percent conservation of pathogenic variant positions in zebrafish genes, as defined in Table 2. Conservation is reported in amino acid identity **(A)** and similarity **(B)**. **(C-D)** Conservation of pathogenic variant positions in each NMDAR domain, grouped as in Figure 1. Homology is reported in identity **(C)**and similarity **(D).** Dashes indicate domains without reported pathogenic variants. Along with the percent of conservation, the total number of identified variants in the human protein is indicated in parentheses in the ‘a’ paralog column in **(C)**.

Using the criteria in Table 2, amino acid positions with pathogenic missense variants in GluN1, GluN2A, GluN2B, and GluN2D are completely conserved in zebrafish paralogues (Fig. 2A–D).

Putatively pathogenic variants are also conserved in these NMDAR subunits, though less so than pathogenic variants. The highest degree of conservation of putatively pathogenic variants is in the GluN1 paralogues (≥ 88% identity; 93% similarity). Putatively pathogenic variants in the GluN2 subunits are also reasonably conserved: zGluN2Aa (70% identity; 92% similarity), zGluN2Ab (62% identity; 84% similarity), zGluN2Ba (65% identity; 71% similarity), zGluN2Bb (72% identity; 80% similarity), and zGluN2Da and zGluNDb (≥ 46% identity; ≥59% similarity) (Fig. 2E,F). In general, the highest level of conservation is in the TMD and TMD-LBD linkers, followed by the LBD, and less so in the NTD and CTD (Fig. 2G,H).

### Benign and putatively benign variant positions show reduced conservation

**Figure 3:**
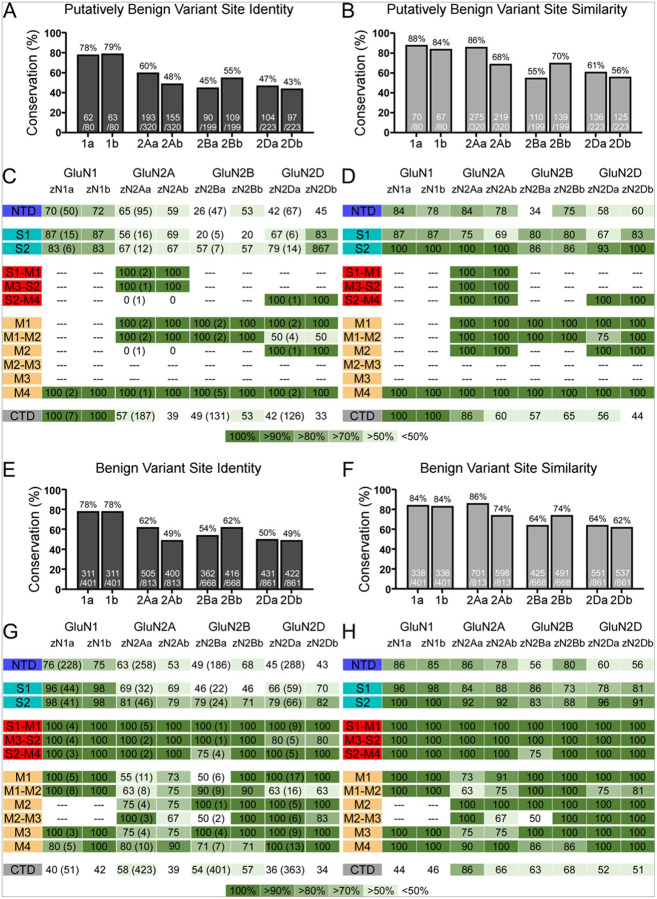
Conservation of positions with putatively benign and benign missense variants **(A-B)** Percent conservation of putatively benign variant positions in zebrafish genes, as defined in Table 2. Conservation is reported in identity **(A)** and similarity **(B)**. **(C-D)** Conservation of putatively benign variant positions in each NMDAR domain, grouped as in Figure 2. Homology is reported in identity **(C)** and similarity **(D)**. Dashes indicate domains without reported pathogenic variants. Along with the percent of conservation, the total number of identified variants in the human protein is indicated in parentheses in the ‘a’ paralog column in **(C)**. **(E-H)** As in A-D but for benign variant positions.

We carried out a comparable analysis as above for benign and putatively benign variants (Table 2). We hypothesized that positions with more benign variants would be less well conserved than pathogenic ones. Indeed, the general trend is consistent with this idea (Fig. 3). Putatively benign variants showed a reduced conservation compared to putatively pathogenic variants (putatively pathogenic vs putatively benign): zGluN1a (88% vs. 78% identity; 93% vs. 88% similarity), zGluN1b (89% vs. 79% identity; 93% vs. 84% similarity), zGluN2Aa (70% vs. 60% identity; 92% vs. 86% similarity), zGluN2Ab (62% vs. 48% identity; 84% vs. 68% similarity), zGluN2Ba (65% vs. 45% identity; 71% vs. 55% similarity), zGluN2Bb (72% vs. 55% identity; 80% vs. 70% similarity), zGluN2Da (46% vs. 47% identity; 59% vs. 61% similarity), and zGluN2Db (47% vs. 44% identity; 61% vs. 56% similarity) (Fig. 2E,F vs Fig. 3A,B). For these putatively benign variants, the greatest extent of conservation tends to occur in the TMD and LBD-TMD linkers, followed by the LBD, and then the NTD and CTD (Fig. 3C,D).

For benign variants, however, the decrease in conservation plateaus, with identity and similarity values comparable to the putatively benign variants, if not slightly higher (Fig. 3E–F). The subdomain-specific conservation trend for these variants follows that seen on the level of the whole receptor (Fig. 1), wherein the TMD, LBD-TMD linkers, and LBD are generally more conserved and the NTD and CTD less so (Fig. 3G,H). Although diverging from our prediction as we do not note a substantial decrease in the conservation of benign variants with respect to the putatively benign variants, this result reflects the overall conservation of the NMDAR subunits themselves (benign vs total protein): zGluN1 (78% vs. ≥84% identity; 84% vs. ≥89% similarity), zGluN2Aa (62% vs. 68% identity; 86% vs. 89% similarity), zGluN2Ab (49% vs. 58% identity; 74% vs. 79% similarity), zGluN2Ba (54% vs. 65% identity; 64% vs. 73% similarity), and zGluN2Bb (62% vs. 67% identity; 74% vs. 81% similarity), zGluN2Da (50% vs. 43% identity; 64% vs. 54% similarity), and zGluN2Db (49% vs. 46% identity; 62% vs. 57% similarity) (Fig. 3E–F, Table 1). Furthermore, this result reflects the relatively large number of benign variants in comparison to other variant groups (Table 2).

### Critical TMD and LBD-TMD linker components are completely conserved

Elements forming the ion channel – the TMD (M1, M1-M2, M2, M2-M3, M3, & M4) – and the LBD-TMD linkers (S1-M1, M3-S2, & S2-M4) were consistently highly conserved (Fig. 1). Nevertheless, we assayed conservation of critical functional motifs. The S1-M1 linker is a critical element for channel gating and is completely conserved (Fig. S1) (56). The M2 subdomain contains what is known as the ‘N’ and ‘N + 1’ sites, which participate in Mg^2+^ block and Ca^2+^ permeation through the channel (2, 55) and are conserved in zebrafish (Fig. S1, dark orange). The TMD M3 segment contains the SYTANLAAF motif, which plays a key role in ion channel gating and is known to be highly conserved across species. This motif is conserved in all zebrafish GluN1 and GluN2 paralogs (Fig. S1, orange). Lastly, the ‘DRPEER’ motif in M3-S2 of GluN1, an important component in the high Ca^2+^ permeability of NMDARs (52) is conserved (Fig. S1, light orange). Hence, core gating and permeation elements of the TMD and associated regions are highly conserved suggesting that basic mechanisms of ion channel gating and block/permeation are preserved.

### LBD elements required for agonist binding are completely conserved

The GluN1 LBD binds glycine or D-serine while the GluN2 LBD binds glutamate or aspartate (Fig. S2A,B). Overall, the LBDs are highly conserved in zebrafish relative to humans (Fig. 1), as are specific amino acid side chains in S1 and S2 that are involved in binding endogenous agonists (47) (Fig. S2C). These include sites that participate in Van-der Waals interactions with the α-carbon of the ligand (darkest red), interactions with the α-amino group (dark red), electrostatic interactions with the α-carboxyl group (red), and interactions with the amino acid side chain (light red) (Fig. S2C).

### Conservation of structure-function elements in the NTD

In terms of overall sequence comparisons, the NTD shows variable degrees of conservation across NMDAR subunits. The GluN1 zebrafish paralogs maintain the highest degree of conservation relative to human (80% identity; ≥88% similarity) (Fig. 1). The GluN2 subunits show decreased NTD conservation (49–75% identity; 59–88% similarity) (Fig. 1). Given this reduced conservation, we assayed the conservation of key structure-function motifs in zebrafish paralogues.

**Figure 4:**
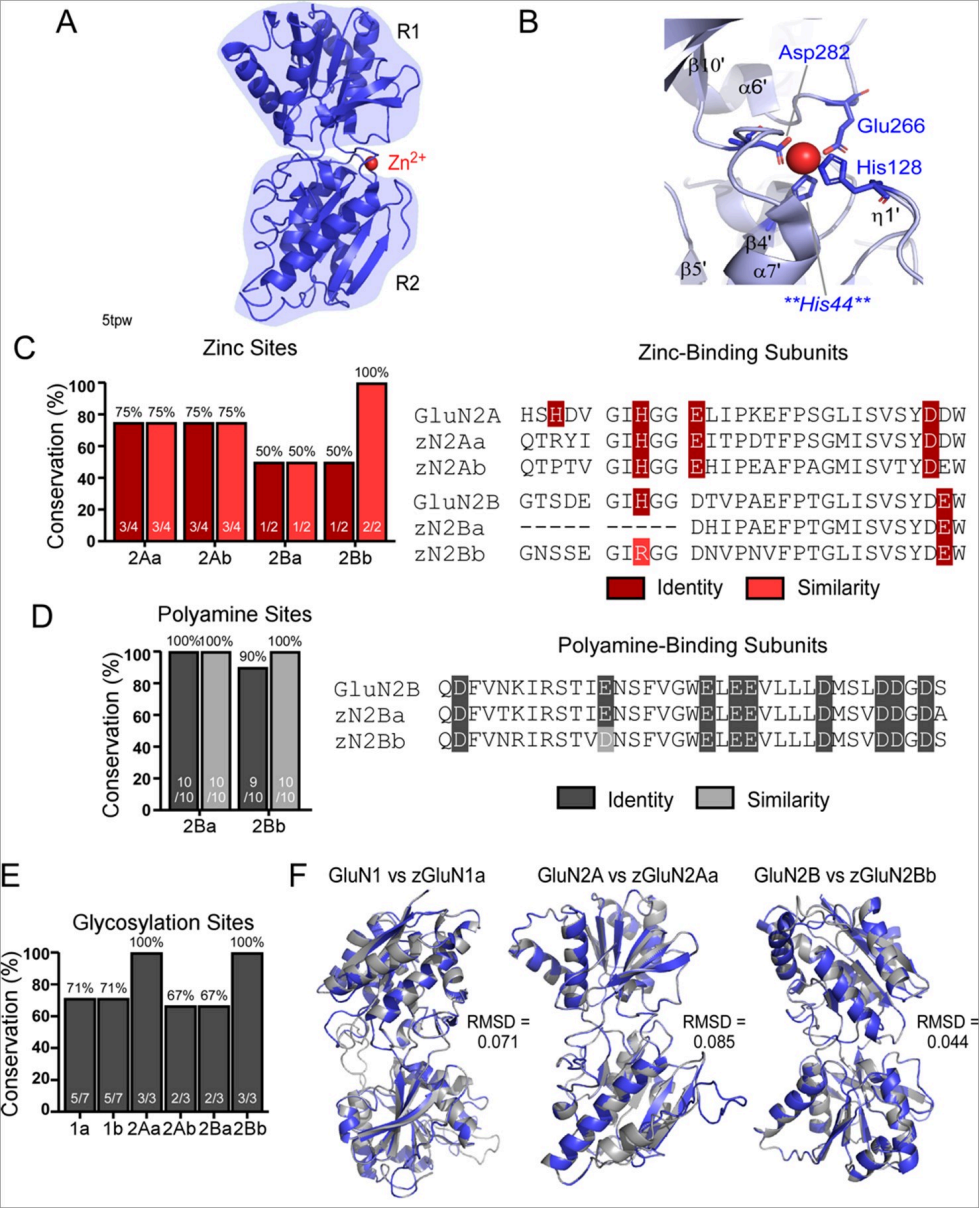
Conservation of key functional elements and structure of the NMDAR NTD **(A)** Topology of GluN2A NTD, showing R1 and R2 subdomains, bound to zinc (red). 5tpw (58) **(B)** Close-up image of structure in **(A)**highlighting amino acids that participate in zinc binding. His44 is demarcated as it is not conserved in the zebrafish paralogues. **(C)** Conservation of amino acids that participate in NTD zinc binding in GluN2A and GluN2B, reported in identity (dark red) and similarity (light red). Sequences are shown to the right. **(D)** Conservation of GluN2B NTD regions that participate in interactions with polyamines, reported in identity (dark gray) and similarity (light gray). Sequences are shown to the right. **(E)** Conservation of NTD glycosylation sites, reported in identity (dark gray). **(F)** Aligned NTD homology models of the human (blue) and zebrafish (gray) protein for GluN1 vs zGluN1a (reference structure, 5tp0) (*left*), GluN2A vs zGlun2Aa (reference structure, 5tp0) (*middle*), and GluN2B vs zGluN2Bb (reference structure, 5tpz) (*right*). Secondary structures colored both blue and gray indicate areas of structural overlap.

Among its many roles, the NTD participates in interactions with ions and molecules that modulate receptor activity (58, 59) (Fig. 4A,B). This is most notable for GluN2A and to a lesser extent GluN2B as they bind zinc, which inhibits receptor function by reducing channel open probability (58). In GluN2A, four amino acids coordinate zinc binding: GluN2A-His44, GluN2A-His128, GluN2A-Glu266, and GluN2A-Asp282 (58). Of these key sites, three are conserved (Fig. 4C) but in zGluN2Aa and zGluN2Ab, a key residue involved in Zn^2+^ binding, GluN2A-His44, is not conserved (Fig. 4C). For GluN2B, only two amino acid residues participate in zinc binding: GluN2B-His127 and GluN2B-Glu284 (58). While GluN2B-Glu284 is conserved, the GluN2B-His127 position in zGluN2Ba is missing due to alterations in NTD sequence length leading to gaps in the alignments, though it is similar now as a positively charged arginine instead of a histidine in zGluN2Bb (Fig. 4C).

The NTD of GluN2B also binds polyamines, which enhance receptor activity (59). Nine amino acids are involved in the binding of polyamines. These sites in zGluN2Ba and zGluN2Bb are completely conserved except for one amino acid in zGluN2Bb that is similar but not identical, now a negatively charged aspartate instead of glutamate (Fig. 4D). Most of this functional component of GluN2B is, thus, conserved in zebrafish.

Additionally, the NMDAR NTD has glycosylation sites, and their glycosylation regulates the trafficking of NMDARs to the cell membrane (60). Given the consensus sequence for arginine (N-) glycosylation (N-*X*-S/T), GluN1 has seven, GluN2A has three, and GluN2B has three putative glycosylation sites in the NTD (60). Their conservation in the zebrafish paralogues is somewhat variable: zGluN1a & zGluN1b (5 of 7 sites), zGluN2Aa (3 of 3 sites), zGluN2Ab (2 of 3 sites), zGluN2Ba (2 of 3 sites), and zGluN2Bb (3 of 3 sites) (Fig. 4E). To the best of our knowledge, specific glycosylation sites in GluN2D have yet to be identified. Of note, the GluN1 glycosylation sites GluN1-N203 and GluN1-N368 are necessary for receptor surface expression (60), and these sites are conserved in the zebrafish paralogues.

While the critical functional features of the LBD were completely conserved in zebrafish relative to human NMDARs, the conservation of key regions in the NTD occasionally diverged, most notably that for Zn^2+^ binding. To further explore the structural composition of the GluN1, GluN2A, and GluN2B NTDs, we used SWISS-MODEL to generate homology models for the human and zebrafish NMDAR subunits, which we subsequently aligned in PyMole to assess conservation at the level of the three-dimensional structure (see Methods) (Fig. 4F). In general, this analysis revealed a high degree of similarity between the human NTD and zebrafish paralogs. The zebrafish GluN1 NTD models display similar overall topology to human: GluN1 vs zGluN1a, reference structure 5tp0 (RMSD = 0.071); GluN1 vs zGluN1a, reference structure 5tpz (RMSD = 0.085); GluN1 vs zGluN1b, reference structure 5tp0 (RMSD = 0.094); and GluN1 vs zGluN1b, reference structure 5tpz (RMSD = 0.097) (Table S3).

The GluN2A and GluN2B subunits also reflect a high degree of structural similarity: GluN2A vs zGluN2Aa, reference structure 5tp0 (RMSD = 0.085); GluN2A vs zGluN2Ab, reference structure 5tp0 (RMSD = 0.095); GluN2A vs zGluN2Ab, reference structure 5tpw (RMSD = 0.111); GluN2B vs zGluN2Ba, reference structure 5tpz (RMSD = 0.028); and GluN2B vs GluN2Bb, reference structure 5tpz (RMSD = 0.044) (Table S3). The only exception here is the structural comparison of GluN2A and zGluN2Aa using reference structure 5tpw, in which the NTD is interacting with zinc (RMSD = 2.149), suggesting that the structural components of zGluN2Aa may not effectively complex with zinc. Interestingly, zGluN2Ba is > 100 amino acids shorter and zGluN2Bb is > 70 amino acids longer than human GluN2B. Though the zGluN2Ba model is missing an α-helix relative to the human protein, the zGluN2Bb modelled structure aligns well with its human counterpart, suggesting that the zebrafish sequences maintain a large degree of structural integrity.

### Conservation of post-translational modification (PTM) sites in the CTD

The intracellular CTD is a key regulatory domain of NMDAR cell biology and function and shows distinct subunit-specific elements (11). For our analyses, we focused on the GluN1–1 CTD splice variant, which is the longest variant containing the C0-C1-C2 cassettes (11).

Across the NMDAR subunits, the zebrafish CTDs are the most poorly conserved (Fig. 1). With a sequence identity generally around 50–60% for the GluN1, GluN2A, and GluN2B zebrafish paralogs, with some exceptions: zGluN1a (53% identity; 58% similarity), zGluN1b (54% identity; 59% similarity), zGluN2Aa (57% identity; 86% identity), zGluN2Ab (38% identity; 65% similarity), zGluN2Ba (57% identity, 66% similarity), and zGluN2Bb (60% identity, 71% similarity) while that of GluN2D is much lower (≥ 18% identity, ≥ 28% similarity) (Fig. 1).

**Figure 5:**
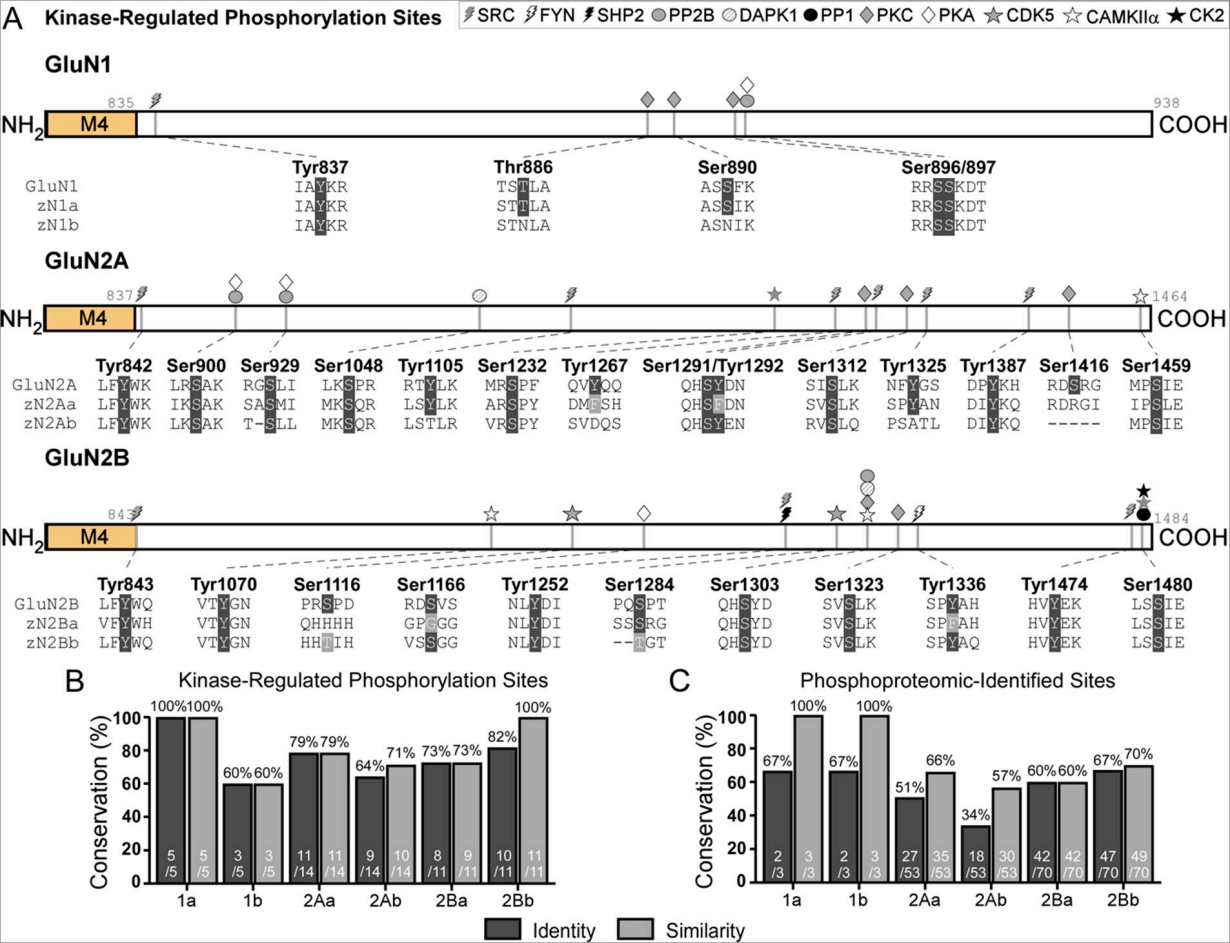
Conservation of key NMDAR CTD phosphorylation sites **(A)** Schematics of CTD kinase-regulated phosphorylation sites for the GluN1, GluN2A and GluN2B CTDs. The specific phosphorylation sites and the corresponding human and zebrafish sequences are indicated. Positions sharing identity are indicated in dark gray. Similarity is shown in light gray; here, a residue is only considered similar if it is ‘phosphorylatable’ (e.g., contains a S, T, or Y) (see Methods). **(B-C)** Percent conservation of kinase-regulated phosphorylation sites **(B)** and other phosphorylation sites identified by phosphoproteomics **(C)**. Conservation is reported in identity (dark gray) and similarity (light gray); similarity is defined in **(A)**.

Given these deviations in protein sequences, we first examined the conservation of CTD regions critical for post-translational modifications (PTMs), excluding GluN2D due its poor conservation in zebrafish. Phosphorylation of NMDAR subunit CTDs confers key functional roles to the receptor (2, 11). Initially, we examined experimentally validated sites at which phosphorylation occurs by known kinases and calculated the percent conservation, reported in identity and similarity. Similarity, here, is defined as a site at the homologous position that can be phosphorylated (e.g., S, T, or Y), if not identical. The GluN1 CTD contains phosphorylation sites for SRC kinase (SRC), protein kinase C (PKC), and protein kinase A (PKA), (Fig. 5A, *top*) (11). Note specific sites have also been identified that are also targeted by the serine/threonine protein phosphatase 2B (PP2B). These PTM sites are perfectly conserved in zGluN1a, while three of five are conserved in zGluN1b (60% identity & similarity) (Fig. 5B). The GluN2A CTD is phosphorylated by SRC, PKA, Dual specificity tyrosine-phosphorylation-regulated kinase 1A (DYRK1), cyclin-dependent kinase 5 (CDK5), PKC, and calcium/calmodulin-dependent protein kinase IIα (CaMKIIα) (Fig. 5A, *middle*) (11): zGluN2Aa (79% identity & similarity) and zGluN2Ab (64% identity, 71% similarity) (Fig. 5B). The CTD of GluN2B is phosphorylated by SRC, CaMKIIα, CDK5, PKA, PKC, death-associated protein kinase 1 (DAPK1), Proto-oncogene tyrosine-protein kinase Fyn (FYN), and Casein Kinase II (CK2) (Fig. 5A, *bottom*) (11). GluN2B also contains sites for the protein tyrosine phosphatase non-receptor 11 (PTPN11, or SHP2) and protein phosphatase 1 (PP1). The conservation of these PTMs is slightly higher than that in GluN2A: zGluN2Ba (73% identity & similarity) and zGluN2Bb (82% identity, 100% similarity) (Fig. 5B).

In addition to these kinase-regulated sites, we also examined the conservation of other known phosphorylation sites in these subunits identified by phosphoproteomics (PSP) (see Methods). The conservation of these sites in the zebrafish paralogs was generally lower than that of the kinase-regulated sites: GluN1 (67% identity, 100% similarity), zGluN2Aa (51% identity, 66% similarity), zGluN2Ab (34% identity, 57% similarity), zGluN2Ba (60% identity & similarity), and zGluN2Bb (67% identity, 70% similarity) (Fig. 5C).

**Figure 6:**
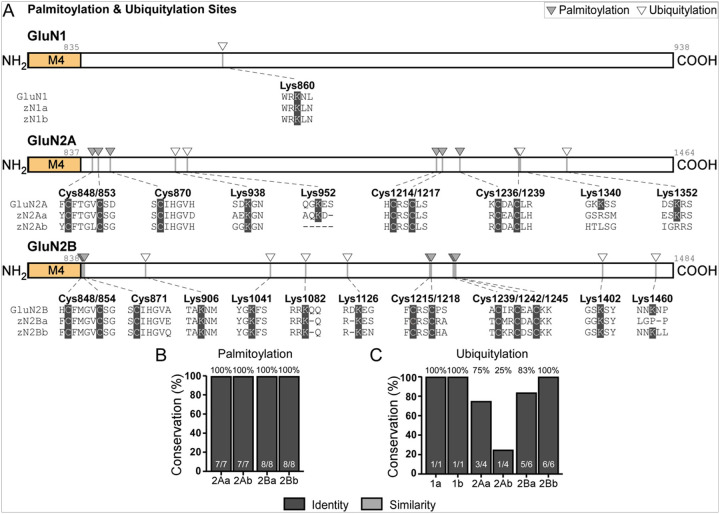
Conservation of key NMDAR CTD post-translational modification (PTM) sites **(A)** Schematics of CTD palmitoylation and ubiquitylation sites for the GluN1, GluN2A and GluN2B CTDs. The specific phosphorylation sites and the corresponding human and zebrafish sequences are indicated. Positions sharing identity are indicated in dark gray. **(B-C)** Percent conservation of palmitoylation **(B)** and ubiquitylation **(C)** sites, reported in identity (dark gray).

We next examined the conservation of CTD palmitoylation and ubiquitylation sites. GluN1 has a single ubiquitylation site (Lys860) (Fig. 6A, *top*) (11), and it is conserved in the zebrafish paralogs (Fig. 6C). GluN2A contains seven palmitoylation sites (Fig. 6A, *middle*) (11), which are likewise completely conserved in zebrafish (Fig. 6B). The GluN2A CTD also has four ubiquitylation sites (Fig. 6A, *middle*) (11), of which three are conserved in zGluN2Aa (75%) and one in zGluN2Ab (25%) (Fig. 6C). With the same approach for GluN2B, we identified eight palmitoylation and six ubiquitylation sites (Fig. 6A, *bottom*) (11). Here, all palmitoylation sites are conserved in zebrafish while five of six ubiquitylation sites (83%) are conserved in zGluN2Ba and all six ubiquitylation sites (100%) are conserved in zGluN2Bb (Fig. 5B,C).

In summary, the global conservation of PTM sites is as follows: zGluN1a and zGluN1b (89% identity, 100% similarity), zGluN2Aa (62% identity, 72% similarity), zGluN2Ab (45% identity, 62% similarity), zGluN2Ba (66% identity, 67% similarity), and zGluN2Bb (75% identity, 78% similarity).

### Conservation of protein-binding motifs (PBM) in the CTD

In addition to numerous PTM sites, a variety of protein-protein interactions are critical to the role of the CTD in receptor cell biology (surface expression, distribution) and function (11, 63). These protein-binding elements include either defined short linear motifs (SLiMs) or broader regions known to serve as protein docking sites.

**Figure 7:**
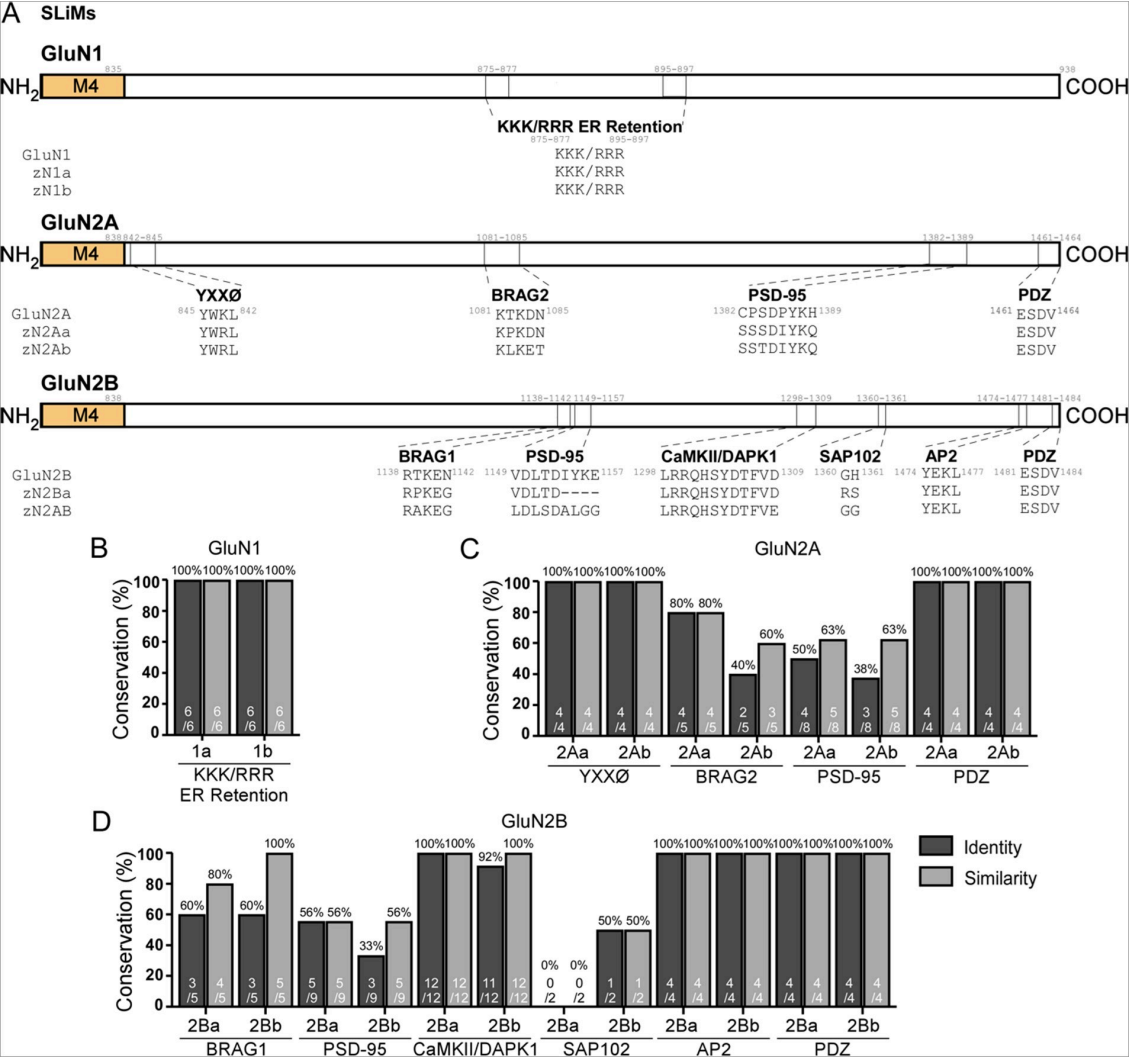
Conservation of NMDAR short linear motif (SLiM) protein-binding motifs (PBMs) in the CTD **(A)** Schematics of CTD short linear motifs (SLiMs) for the GluN1, GluN2A and GluN2B CTDs. **(B-D)** Percent conservation of CTD SLiMs in GluN1 **(B),** GluN2A **(C),** and GluN2B **(D)**. Conservation is reported in identity (dark gray) and similarity (light gray).

**Figure 8:**
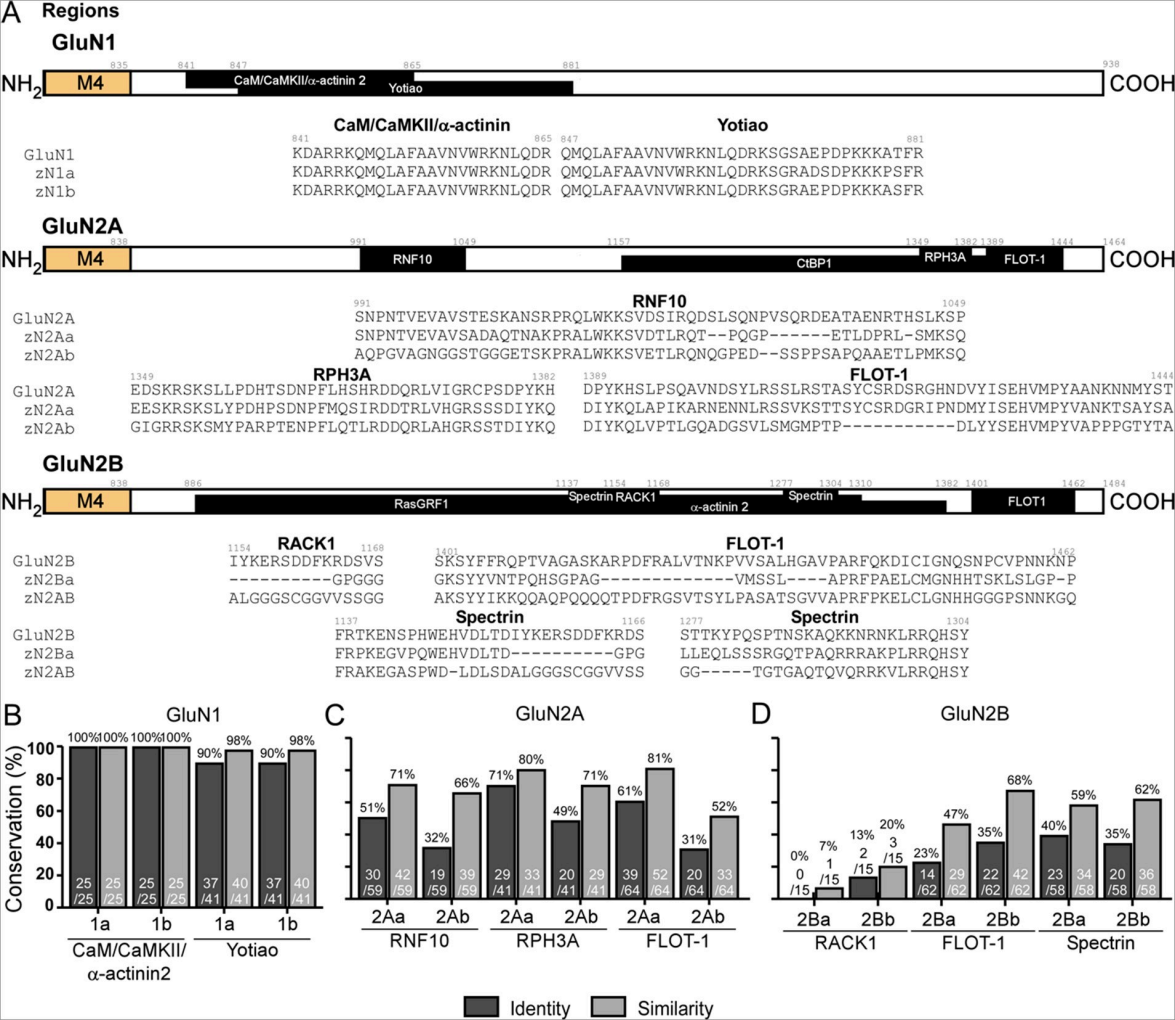
Conservation of NMDAR protein-binding motifs (PBMs) regions in the CTD **(A)** Schematics of CTD regions for the GluN1, GluN2A and GluN2B CTDs. **(B-D)** Percent conservation of CTD regions in GluN1 **(B)**, GluN2A **(C)**, and GluN2B **(D)**. Conservation is reported in identity (dark gray) and similarity (light gray).

The CTD of GluN1 contains a SLiM known as the KKK/RRR ER retention motif, which is conserved in zebrafish (66) (Fig. 7A,B). It, likewise, has binding regions for calmodulin (CaM)/calcium/calmodulin-dependent protein kinase II (CaMKII) and α-actinin 2 (64, 65, 67, 78), which is conserved and Yotiao, also known as A-kinase anchoring protein 9 (AKAP9) (67), which is generally well conserved: zGluN1a (90% identity, 98% similarity) and zGluN1b (90% identity, 98% similarity) (Fig. 8A,B).

The GluN2 subunit CTDs contain a variety of PBMs. For GluN2A, the conservation of SLiMs is: the YXXØ motif, where Y is tyrosine, X is any amino acid, and Ø is a bulky hydrophobic residue like leucine, isoleucine, phenylalanine, methionine, or valine (73) – both zGluN2Aa and zGluN2Ab show 100% identity & similarity; the guanine-nucleotide exchange factor BRAG2 (70) – zGluN2Aa (80% identity & similarity) and zGluN2Ab (40% identity, 60% similarity); postsynaptic density protein 95 (PSD-95) (72) – zGluN2Aa (50% identity; 63% similarity) and zGluN2Ab (38% identity, 63% similarity); and the PDZ domain that interacts with the proteins PSD-95, discs large (Dlg), and zonula occludens-1 (ZO-1) (11) – zGluN2Aa and zGluN2Ab (100% identity & similarity) (Fig. 7A,C). Interacting regions in GluN2A include: Ring Finger Protein 10 (RNF10) (70) – zGluN2Aa (51% identity, 72% similarity) and zGluN2Ab (32% identity, 66% similarity); Rabphilin 3A (RPH3A) (68) – zGluN2Aa (71% identity, 80% similarity) and zGluN2Ab (49% identity, 71% similarity); Flotillin-1 (FLOT-1) (71) – zGluN2Aa (61% identity, 81% similarity) and zGluN2Ab (31% identity, 52% similarity); and C-terminal binding protein 1 (CtBP1) (69) – zGluN2Aa (51% identity, 85% similarity) and zGluN2Ab (36% identity, 63% similarity) (Fig. 8A,C, S3A,B).

For GluN2B, the conservation of SLiMs is: the guanine-nucleotide exchange factor BRAG1 (76) – zGluN2Ba (60% identity; 80% similarity) and zGluN2Bb (60% identity, 100% similarity); PSD-95 (72) – zGluN2Ba (56% identity & similarity) and zGluN2Bb (33% identity, 56% similarity); CaMKII and death-associated protein kinase 1 (DAPK1) (11) – zGluN2Ba (100% identity & similarity) and zGluN2Bb (92% identity, 100% similarity); synapse-associated protein 102 (SAP102) (75) – zGluN2Ba (0% identity & similarity) and zGluN2Bb (50% identity & similarity); the adaptor complex AP2 (74) – zGluN2Ba (100% identity & similarity) and zGluN2Bb (100% identity & similarity); and PDZ (11) – zGluN2Ba (100% identity & similarity) and zGluN2Bb (100% identity & similarity) (Fig. 7A,D). The CTD interaction regions for GluN2B are conserved as follows: receptor for activated C kinase 1 (RACK1) (77) – zGluN2Ba (0% identity, 7% similarity) and zGluN2Bb (13% identity, 20% similarity); FLOT-1 (71) – zGluN2Ba (23% identity; 47% similarity) and zGluN2Bb (35% identity, 68% similarity); Spectrin (79) – zGluN2Ba (40% identity; 59% similarity) and zGluN2Bb (35% identity, 62% similarity); α-actinin 2 (78) – zGluN2Ba (47% identity; 67% similarity) and zGluN2Bb (51% identity, 76% similarity); and Ras protein-specific guanine nucleotide-releasing factor 1 (RasGRF1) (80) – zGluN2Ba (59% identity; 74% similarity) and zGluN2Bb (60% identity, 78% similarity) (Fig. 8A,D, S3A,C).

When compared globally across subunits, SLiMs always show greater conservation than regions. This conservation is always greatest in the zebrafish GluN1 paralogs, followed by the GluN2B and then GluN2A paralogs. For the analyzed SLiMs, conservation grouped across each subunit is as follows: zGluN1a and zGluN1b (100% identity & similarity), zGluN2Aa (76% identity, 81% similarity), zGluN2Ab (62% identity, 76% similarity), zGluN2Ba (78% identity, 81% similarity), and zGluN2Bb (72% identity, 86% similarity). The regions show the following degrees of conservation: zGluN1a and zGluN1b (94% identity, 98% similarity), zGluN2Aa (55% identity, 81% similarity), zGluN2Ab (36% identity, 62% similarity), zGluN2Ba (59% identity, 74% similarity), and zGluN2Bb (60% identity, 78% similarity).

## DISCUSSION

Here, we compared the conservation of the zebrafish NMDAR subunit paralogues, including key structure-function motifs, to their human counterparts. We find that the core gating machinery – the LBD, the LBD-TMD linkers and the TMD – are highly conserved both in terms of overall identity and specific functional motifs. The more peripheral domains – the NTD and CTD – show less conservation overall, though functional motifs are reasonably conserved with the notable exception of Zn^2+^ binding in the GluN2A NTD. Notably, disease-associated variants are generally conserved, highlighting that zebrafish represent a useful model to study NMDARs.

### NMDAR conservation in zebrafish

Across most of our examined parameters, the obligatory GluN1 subunit shows the highest degree of conservation in zebrafish (Fig. 1). Generally, conservation is then greatest in the GluN2(A-D) followed by the GluN3(A-B) subunits. Within each subunit, sequence conservation is consistently highest in the TMD and LBD-TMD linkers, followed by the LBD. Conservation is generally poorer in the NTD and even worse in the CTD (Fig. 1).

A key question with regards to this work is defining what constitutes as a ‘well conserved’ sequence. Previous studies regarding the conservation of protein structure and function suggest that sequence identities between 40–70% confer functional conservation (92–94). They regard 50% sequence identity as the threshold below which function drastically diverges (93, 94). Our sequence conservation analyses almost always led to sequence identities > 50%, except for zGluN2Da. Nevertheless, we carried out more detailed examinations of more poorly conserved domains, e.g., the NTD and CTD, to assess the conservation of key structure-function motifs.

### NMDAR missense variant conservation in zebrafish

To date, a multitude of variants, including missense, frameshift, and nonsense mutations, have been identified in the genes encoding the NMDAR subunits (21, 22, 87). We categorized identified missense variants by pathogenicity and analyzed the extent of conservation of the positions at which they occur. We focused only on the GluN1, GluN2A, GluN2B, and GluN2D subunits for this analysis, as this is where most disease-associated variants have been identified. Generally, our results demonstrate a higher degree of variant position conservation with increasing pathogenicity (Fig. 2,3). The sites of pathogenic missense variants are almost perfectly conserved (Fig. 2). Given the high conservation of the amino acid positions at which they occur, these protein regions are likely most essential for receptor function. Across our categories of variants, with the exclusion of complex variants, the proportion of the total protein at which missense variants have been identified is lowest in the pathogenic group (Table 2). It is possible that additional variants that might be categorized as pathogenic are so devastating to development as to be embryonic lethal. This reflects the notion that more integral regions of the receptor are less tolerant to variants (95). Zebrafish, nevertheless, emerge as a useful model for the study of pathogenic NMDAR variants.

Putatively pathogenic variants are conserved to a lesser extent than their pathogenic counterparts (Fig. 2). According to our designations, these variants have the potential to cause disease, having been identified in patients, but are currently classified as having uncertain significance. Thus, it aligns that these variants are comparatively less well conserved – they are likely occurring in less critical regions of the receptor and are likewise less likely to cause disease phenotypes.

Our subsequent investigation of putatively benign and benign variants revealed a further decrease in variant position conservation. This finding further supported our prediction that variants more highly associated with disease occur at positions with higher degrees of conservation and, thus, are more critical to protein structure and function. The conservation of positions with putatively benign and benign variants is quite comparable. For the benign variants, in particular, conservation percentages also closely reflect total protein conservation values for each paralog (Fig. 3, Table 1). This follows from the finding that positions with identified benign variants, for most subunits, make up a substantial portion of the protein (Table 2).

Nevertheless, this investigation both demonstrates the advantage of the zebrafish model in studying NMDAR-associated disease and reflects the notion that the positions most essential for receptor function are those that are best conserved and more likely to induce disease when absent.

Overall, the trend for variant conservation is the same as that for the whole protein. Conservation is always greatest in the TMD and LBD-TMD linkers, followed by the LBD, next the NTD, and last the CTD (Fig. 2, 3). These findings suggest that zebrafish serve as an effective model for variants, especially pathogenic and putatively pathogenic variants in both the TMD and LBD-TMD linkers. Some caution, however, must be exercised when using this model system for variants in the LBD, NTD, and CTD.

### TMD, TMD-LBD linker, and LBD conservation

Our examination of key functional regions and motifs in the TMD, LBD-TMD linkers, and LBD consistently revealed complete conservation in their respective zebrafish paralogs (Fig. S1,S2). This suggests that the basic mechanism of ion channel gating is conserved in zebrafish relative to human. As these domains and subdomains contribute to the formation of the core of the receptor – the ion channel pore allowing for ion flux – it is expected that they would be most highly conserved across species, since the major functional role of NMDARs is current flux during synaptic transmission.

### NTD conservation

The NTD is the first region in which NMDAR sequences in zebrafish begin to diverge from those in human. Modelling NTD variants in zebrafish must, thus, be approached with caution. For example, polyamine-binding sites in GluN2B are almost completely conserved in zebrafish (Fig. 4D), so they have the potential to serve as an effective model for this functional role of NMDARs. On the other hand, zinc-binding sites in GluN2A and GluN2B are more poorly conserved in zebrafish (Fig. 4C), so they would likely serve as a poor model for zinc-related studies in NMDARs. This also aligns with our homology model finding that the zGluN2Aa model aligns well with GluN2A when not bound to zinc, but the RMSD of the alignment is substantially poorer when GluN2A interacting with zinc is used as the reference structure (Table S3).

### CTD conservation

The C-terminal domains (CTDs) exhibit the lowest level of overall conservation across NMDAR domains (Fig. 1). This is consistent with the CTD’s classification as an intrinsically disordered region (IDR), which lacks fixed secondary or tertiary structure and is more tolerant to evolutionary divergence (96, 97). Nonetheless, functional conservation within IDRs is often maintained through the retention of post-translational modification (PTM) hotspots and short linear motifs (SLiMs) that regulate protein interactions and intracellular signaling (98).

We assessed CTD conservation in GluN1, GluN2A, and GluN2B by analyzing experimentally validated phosphorylation, palmitoylation, and ubiquitylation sites, as well as defined protein-binding motifs. Phosphorylation sites with known kinase interactions exhibited relatively high conservation, consistent with their well-characterized regulatory roles (Figs. 5A,B). In contrast, phosphorylation sites without identified kinases were less consistently retained (Fig. 5C). Palmitoylation sites were fully conserved across all subunits, while ubiquitylation sites were generally retained, though conservation was notably lower in zGluN2Ab (Fig. 5F) and may reflect paralogue-specific divergence in degradation or trafficking pathways.

Protein-binding motif conservation varied by subunit. GluN1 retained nearly all canonical interaction motifs, while GluN2A and GluN2B displayed region-specific conservation. Notably well-conserved elements in the GluN2 subunits included the PDZ-binding motifs as well as docking regions for CaMKII, DAPK1, and AP2 (Fig. 6). SLiMs overall showed higher conservation, likely due to their short, sequence-specific structure, which may place them under stronger evolutionary constraint than broader, more flexible interaction regions.

Despite broad sequence divergence, NMDAR CTDs retain a core set of conserved motifs involved in receptor trafficking, synaptic localization, and plasticity. Kinase-regulated phosphorylation sites, palmitoylation sites, and PDZ-binding motifs were consistently preserved. Conservation of GluN1 sites was always greatest in the zebrafish paralogs. Among the GluN2 subunits, GluN2B displayed the highest overall conservation of functional elements, aligning with its essential role in neurodevelopment and synaptic signaling.

These findings are especially relevant to the study of disease-associated truncating variants in the NMDAR subunits. Nonsense variants that introduce premature stop codons in the CTD eliminate key regulatory motifs critical for trafficking, anchoring, or signaling. When these affected regions correspond to conserved functional elements, zebrafish may serve as an appropriate *in vitro* and *in vivo* model for studying CTD-mediated disease mechanisms. Conversely, for mutations in poorly conserved regions, the translational utility of this model may become limited.

## CONCLUSIONS

Zebrafish represent a valuable system to study NMDAR-associated neurodevelopmental disorders, but one must be judicious in choosing which variants to study in this model. Our unique approach of sequence alignments per individual NMDAR domains and subdomains highlights the high degree of conversation of some regions of the protein relative to others. Additionally, our analysis of identified variants indicates that zebrafish are an effective model for pathogenic variants as these occur at conserved locations along the protein. Generally, the utility of zebrafish is most apparent for the study of the TMD, LBD-TMD linkers, and LBD as these regions are most well conserved. On the other hand, studying the NTD and CTD in this species requires extra consideration stemming from deviations in sequence conservation. Our work will enable future NMDAR-related studies that can be effectively and efficiently conducted in zebrafish.

## Supplementary Material

Supplementary Files

This is a list of supplementary files associated with this preprint. Click to download.

- zfishNMDARssupplement20250716.docx

## Figures and Tables

**Table 1:** Overall protein conservation in mouse and zebrafish relative to human

	Human	Mouse			Zebrafish paralogue a			Zebrafish paralogue b		
	Protein Length	Id.	Sim.	Protein Length	Id.	Sim.	Protein Length	Id.	Sim.	Protein Length
N1	938	97%	97%	938	84%	89%	966 (−28)	86%	90%	937 (−1)
N2A	1464	95%	98%	1464	68%	89%	1460 (+ 6)	58%	79%	1445 (−9)
N2B	1484	98%	99%	1482 (−2)	65%	73%	1417 (−31)	67%	81%	1770 (+ 322)
N2C	1233	88%	90%	1239 (+ 6)	50%	64%	1400 (−176)	59%	79%	1328 (+ 95)
N2D	1336	97%	97%	1323 (−13)	43%	54%	1916 (+ 580)	46%	57%	1676 (+ 340)
N3A	1115	92%	95%	1135 (+ 20)	63%	78%	1111 (−4)			
N3B	1043	77%	83%	1003 (−40)	54%	62%	1118 (+ 75)	62%	68%	1114 (+ 71)

**Table 2:** Variant designation

			GluN						
Designation	ClinVar	gnomAD	1	2A	2B	2C	2D	3A	3B
Pathogenic	“Pathogenic”	Absent	49 (5%)	58 (4%)	81 (5%)	0	12 (< 1%)	0	0
Putatively Pathogenic	“Uncertain”	Absent	123 (13%)	214 (15%)	182 (12%)	4 (< 1%)	111 (8%)	5 (< 1%)	8 (< 1%)
Complex	“Pathogenic”	Present	3 (< 1%)	5 (< 1%)	6 (< 1%)	0	1 (< 1%)	0	0
Putatively Benign	“Uncertain”	Present	80 (9%)	320 (22%)	199 (13%)	31 (3%)	223 (17%)	30 (3%)	68 (7%)
Benign	“Benign” or Absent	Absent/Present or Present	401 (43%)	813 (55%)	672 (45%)	945 (77%)	863 (27%)	59 (5%)	945 (91%)

## Data Availability

The datasets used and/or analyzed during the current study are available either at OSF (https://osf.io/h2zf9/) or from the corresponding author on reasonable request.

## Abbreviations

NMDAR: *N*-methyl-D-aspartate receptor
NTD: N-terminal domain
LBD: Ligand-binding domain
TMD: Transmembrane domain
CTD: C-terminal domain
